# Repair of type A aortic dissection in a patient with sickle cell disease

**DOI:** 10.1093/jscr/rjaf297

**Published:** 2025-05-13

**Authors:** Jake William duPlooy, Spencer Tingey, Jorge Seoane, Bartlomiej Imielski

**Affiliations:** Department of Cardiothoracic Surgery, Wake Forest University School of Medicine, 1 Medical Center Blvd, 5th Floor Watlington Hall, Winston-Salem, NC 27157, United States; Department of Cardiothoracic Surgery, Wake Forest University School of Medicine, 1 Medical Center Blvd, 5th Floor Watlington Hall, Winston-Salem, NC 27157, United States; Department of Cardiothoracic Surgery, Wake Forest University School of Medicine, 1 Medical Center Blvd, 5th Floor Watlington Hall, Winston-Salem, NC 27157, United States; Department of Cardiothoracic Surgery, Wake Forest University School of Medicine, 1 Medical Center Blvd, 5th Floor Watlington Hall, Winston-Salem, NC 27157, United States

**Keywords:** aortic dissection, sickle cell disease, aortic surgery, aortic root replacement, exchange transfusion, aortic valve surgery

## Abstract

We describe a patient with homozygous HbSS sickle cell anemia and end stage renal disease who presented to our medical center with a Stanford type A aortic dissection. His dissection was successfully managed with a hemiarch repair and concomitant bio-Bentall aortic root replacement. Intraoperatively, he received exchange transfusion with omittance of cell saver. Postoperatively, he remained free of sickling events with a very low HbS fraction. His fluid status was managed by continuation of hemodialysis. His hospital course was otherwise uncomplicated, and he was discharged to home on postoperative Day 6.

## Introduction

Sickle cell anemia (SCD) is the most common hemolytic anemia worldwide, yet the literature on aortic dissection repair in SCD patients remains scarce [1]. We describe our successful management of a patient with SCD and end stage renal disease (ESRD) who presented with a Stanford type A aortic dissection.

## Case report

We present a 27-year-old male with hypertension, seizures, and HbSS anemia complicated by ESRD on hemodialysis (HD), chronic pain, previous acute chest syndrome, and pulmonary hypertension. Surgical history included cholecystectomy and craniotomy with evacuation of hemorrhage. Additionally, he had known aortic root and ascending aortic dilatation.

The patient presented with back and chest pain suspected to be sickle cell pain crisis. Labs revealed acute-on-chronic anemia with hemoglobin of 6.2 (baseline around 7). CT angiography revealed type A dissection with ascending aortic dilatation to 5.3 cm. There was no evidence of organ malperfusion or neurological deficits.

Intra-operative transesophageal echocardiography (TEE) showed normal ventricular function but moderate aortic valve regurgitation. Given his age, a valve sparing root replacement (VSRR) was planned over mechanical valve or root replacement to avoid lifetime anticoagulation and redo root replacement, respectively. Arterial perfusion was instituted by direct aortic cannulation via Seldinger technique into the true lumen using TEE. Given dense uremic adhesions, venous drainage was obtained first percutaneously using Seldinger technique through the left femoral vein, and secondly via the SVC, which was used for retrograde cerebral perfusion. The left ventricle was vented through the right superior pulmonary vein. The patient was cooled to a core temperature of 20.7°C. The aorta was cross-clamped, and the heart was arrested using antegrade Del Nido cardioplegia instilled directly into the coronary ostia.

Inspection revealed dissection extending to the annulus with dilated and thinned sinuses. The non-coronary leaflet of the aortic valve contained a healed perforation suggesting prior endocarditis, preventing VSRR. Instead, bio-Bentall was performed using a 27 mm Edwards Konect Resilia Aortic Valved Conduit graft while cooling.

We routinely cool to 18°C; however, given sickling risks, circulatory arrest was started at 21°C. A 28 mm Hemashield graft was trimmed and anastomosed to the distal hemiarch. Cardiopulmonary bypass (CPB) was resumed and the patient was rewarmed with spontaneous cardioversion. He was separated from CPB in standard fashion. Post-procedure EF was 55% with mildly reduced RV function. The patient was transported to the intensive care unit (ICU) in stable but critical condition.

We planned pre-operatively to utilize exchange transfusion over the patient’s own cell saver blood to reduce sickling risk after protamine administration. Intra-operatively, the patient received 5 L crystalloid, 10 units pRBCs, 20 units cryoprecipitate, 2 units fresh frozen plasma, and 2 units of platelets.

Transfusion threshold was hemoglobin <7 g/dl, which was maintained without further transfusion. HgbS concentration was 2.2%. He continued outpatient hydroxyurea, folate, and Oxbryta as prescribed and remained free from sickling complications.

Post-operative pain management was complicated by opioid tolerance. He received hydromorphone drip in the ICU; upon extubation, he switched to oral oxycodone but reported high pain scores. The acute pain management service provided additional support.

The patient resumed regular HD on POD1 and was discharged home on POD6.

## Discussion

Little has been written on aortic surgery in the SCD patient. However, certain principles have emerged for cardiac surgical management. The foundation remains minimization of conditions that favor sickling: hypoxemia, acidosis, hypovolemia, vasoconstriction, and high HbS. This requires interdisciplinary collaboration.

A hallmark of SCD management in surgical patients is preoperative blood infusion to increase Hb and dilute HbS, as recommended by the American Society of Hematology [2, 3]. However, this was not feasible given the emergent presentation, and we elected for red cell exchange intraoperatively without cell saver. Previous case reports have shown intraoperative exchange transfusion a suitable adjunct to preoperative infusion; less evidence exists for it in isolation. Our excellent HbS result suggests that intraoperative exchange transfusion can adequately manage HbS in patients requiring emergent surgery.

Cooling posed another challenge. Traditional wisdom prescribes normothermia at odds with the deep hypothermic circulatory arrest standard in aortic arch surgery due to the perceived risk of triggering acute sickling [2]. Capoccia presents a patient successfully cooled to 24°C during dissection repair [4]. We cooled our patient further to 21°C without sickling complication, further supporting Crawford’s claim that normothermia may be unnecessary if hypoxia, acidosis, and hypovolemia are avoided [5].

The literature differs regarding selection of mechanical vs bioprosthetic valve in a young SCD patient, with a concern for superior valve longevity balanced against the risk from lifelong anticoagulation. We favored bioprosthetic: the RIETE study found long-term anticoagulation in anemic patients to be independently associated with a significantly increased risk of major bleeding events [6]. The danger of mechanical hemolysis and subsequent sickling in a patient with severe chronic anemia further argued against a mechanical valve.

Postoperative anemia management followed American Association of Blood Banking guidelines for hospitalized SCD patients with a restrictive transfusion threshold of 7 g Hb/dl — also the patient’s baseline [7]. Postoperative fluid management was more problematic, as hypovolemia may precipitate sickling but fluid overload may cause significant pulmonary edema or the lethal acute chest syndrome [5]. Furthermore, SCD strongly predisposes patients to chronic renal dysfunction due to renal sickling; we saw this in our patient as ESRD requiring dialysis. This prompted collaboration with nephrology, who dialyzed as appropriate with achievement of euvolemia.

Adequate acute pain management presents a major challenge, as many SCD patients are tolerant to opioids. This is problematic for patient comfort but also impedes post-operative mobilization and incentive spirometry, which significantly reduces the risk of pulmonary complications in hospitalized SCD patients [8]. It is thus imperative to achieve pain control with a low threshold to engage pain management specialists.

A final takeaway: sickle cell disease and acute aortic dissection are both uncommon diseases, and the frequency of a comorbid presentation is slight. However, our experience shows that it can be successfully managed even in a patient with low baseline functional status. But we must first suspect it. Our patient’s presentation was initially chalked down to pain crisis, overlooking the lethal pathology revealed on imaging. Physicians caring for SCD patients should keep their differentials broad. Sometimes we hear hoofbeats and they really are zebras.
